# Cellular Pre-Adaptation to the High O_2_ Concentration Used in Standard Cell Culture Confers Resistance to Subsequent H_2_O_2_-Induced Cell Death

**DOI:** 10.3390/antiox13030269

**Published:** 2024-02-22

**Authors:** Jack B. Jordan, Miranda J. Smallwood, Gary R. Smerdon, Paul G. Winyard

**Affiliations:** 1 University of Exeter Medical School, Faculty of Life and Health Sciences, University of Exeter, St Luke’s Campus, Exeter EX1 2LU, Devon, UK; jack.jordan@inserm.fr (J.B.J.); m.j.smallwood@exeter.ac.uk (M.J.S.); gary.smerdon@ddrc.org (G.R.S.); 2 DDRC Healthcare, Plymouth Science Park, Research Way, Plymouth PL6 8BU, Devon, UK

**Keywords:** cell culture, oxygen concentration, oxidative stress, hydrogen peroxide, cell death, lipid peroxidation

## Abstract

The addition of hydrogen peroxide (H_2_O_2_) to cultured cells is widely used as a method to modulate redox-regulated cellular pathways, including the induction of programmed cell death in cell culture experiments and the testing of pro- and antioxidant compounds. Here, we assessed the effect on the cellular response to H_2_O_2_ of pre-adapting squamous cell carcinoma cells (A431) to the standard cell culture oxygenation of 18.6% O_2_, compared to cells pre-adapted to a physiological skin O_2_ concentration (3.0% O_2_). We showed that cells pre-adapted to 18.6% O_2_ resisted H_2_O_2_-induced cell death compared to cells pre-adapted to 3.0% O_2_ for 96 h prior to treatment with H_2_O_2_. Moreover, the enzymatic activities of catalase and glutathione reductase, as well as the protein expression levels of catalase, were higher in cells pre-adapted to 18.6% O_2_ compared to cells pre-adapted to 3.0% O_2._ H_2_O_2_-resistant cells, pre-adapted to 18.6% O_2_, exhibited increased nuclear Nrf-2 levels. It is concluded that A431 cells pre-adapted to standard cell culture oxygenation conditions resist H_2_O_2_-induced cell death. This effect may be related to their heightened activation of Nrf-2.

## 1. Introduction

Hydrogen peroxide (H_2_O_2_) has been extensively employed in research laboratory cell culture systems to induce cellular oxidative stress, often in the context of experiments to test the efficacy of antioxidant compounds or to modulate redox-regulated cellular pathways, including the induction of programmed cell death [1,2,3,4,5,6,7,8,9,10,11,12]. Such laboratory-based cell culture experiments using H_2_O_2_ are typically performed under a standard cell culture O_2_ concentration [O_2_] of 18.6% O_2_ [1,2,3,4,5,6,7,8,9,10,11,12]. The value of 18.6% O_2_ is slightly lower than that of atmospheric [O_2_] (20.9%) due to the added partial pressures of water vapour and carbon dioxide (CO_2_) in regular cell culture incubators [13]. However, the physiological O_2_ concentration in vivo (physioxia) ranges from 1.5% in articular cartilage to ~16.5% O_2_ in the upper airway. As such, the majority of in vitro investigations using H_2_O_2_ to induce oxidative stress [1,2,3,4,5,6,7,8,9,10,11,12] have used human cells pre-adapted to an in vitro [O_2_] concentration higher than physioxia [14]. It has been reported that aspects of redox homeostasis change when cells are pre-adapted to standard cell culture oxygenation conditions (i.e., 18.6% O_2_) compared to physioxia [15,16,17,18,19,20]. Moreover, the pre-adaptation of cells to 18.6% O_2_ alters their cellular responses to redox-active treatments such as photodynamic irradiation [15], electrophilic Nrf-2 activators [16,17], copper oxide nanoparticles [18], rotenone [19], glycolic acid [21], gluconolactone [21], and quercetin [21]. However, there is little information about changes (and the mechanisms underlying any such changes) in the cellular responses to H_2_O_2_ after long-term cell culture at 18.6% O_2_ compared to physioxia, despite the widespread use of H_2_O_2_ as an oxidative stress inducer in cell culture-based experiments as mentioned above.

In the present investigation, we tested cellular resistance to H_2_O_2_-induced cell death when using H_2_O_2_ concentrations of a similar order of magnitude to those required to induce mammalian cell death in many previously reported cell culture experiments [3,4,5,6,7,9,11,12]. In contrast to previous studies, we used cells which had been pre-adapted, by culturing over a period of days, to either 18.6% O_2_ or 3.0% O_2_. In our experiments, we used human non-melanoma squamous carcinoma cells, employing a cell line (A431) widely used to test potential redox-active treatments such as new modes of photodynamic irradiation [15]. A431 cells were pre-adapted to 18.6% or 3.0% O_2_ for increasing time periods (24–96 h). Whilst the [O_2_] in human skin can range from 11–6% in the trans-cutaneous layer to 8–2% in the dermal layer [14], an [O_2_] of 3.0% was chosen as it is within the range of the [O_2_] measured in epidermoid carcinoma in vivo [22,23], from which the A431 cells were derived. These cells were then treated with H_2_O_2_, and the extent of cell death was measured. Another endpoint often measured after H_2_O_2_ treatment in mammalian cells is lipid peroxidation [4,24]. As such, the effect of growing cells in 18.6% O_2_ on H_2_O_2_-induced lipid peroxidation compared to cells pre-adapted to 3.0% O_2_ was also measured in the present study. Cumene hydroperoxide (CmOOH), a commonly used inducer of lipid peroxidation [25], was employed to determine whether any such resistance extended to other inducers of lipid peroxidation. GPx is an important antioxidant enzyme which defends against cellular lipid peroxidation [26,27]. As such, the GPx inhibitor mercaptosuccinic acid (MSA; [27]) was used to assess whether cells pre-adapted to 18.6% O_2_ resist lipid peroxidation compared to cells pre-adapted to 3.0% O_2_. Then, the activities of key antioxidant enzymes (CAT, GPx, GR) were also measured in the cells pre-adapted to 18.6% O_2_, compared to the activities measured in the cells pre-adapted to 3.0% O_2_, for increasing lengths of time (24–96 h). Finally, as antioxidant defence is partly regulated by the transcription factor nuclear factor erythroid 2-related factor 2 (Nrf-2) [16,17], we also measured the protein levels of nuclear Nrf-2 in cells pre-adapted to 18.6% O_2_ compared to cells pre-adapted to 3.0% O_2_. Thus, we investigated whether the pre-adaptation of mammalian cells to 18.6% O_2_ conferred them resistance to H_2_O_2_ compared to physioxia, and the potential mechanisms involved in any such resistance.

## 2. Methods

### 2.1. In Vitro Culture of A431 Cells under Standard Cell Culture Oxygen Conditions Compared to Physioxia

A431 cells were obtained from the European Collection of Cell Cultures and were cultured in 4.5 g/L Dulbecco’s Modified Eagle’s Medium (DMEM; Lonza, Slough, England, UK) supplemented with 10% foetal bovine serum (FBS; batch #SH30070.03, Cat #HYC23, US Origin; HyClone, Cramlington, UK), 2% (*v*/*v*) L-glutamine (Lonza) and 2% (*v*/*v*) penicillin/streptomycin (Lonza). Cells were cultured in T75 cm^2^ flasks (#658170; Greiner Bio-One, Stonehouse, UK) for general cell culture, 24-well plates (#3524; Corning, Deeside, UK) for the cell death and lipid peroxidation studies, or 96-well plates (#165305; Thermo Fisher, Loughborough, UK) for the Amplex Red study. Routine passaging was carried out every 3–4 days under aseptic conditions in a class II laminar flow hood. Cells between passages 5 and 10 were used for experimentation. To culture cells in standard cell culture oxygenation conditions (18.6% O_2_), A431 cells were incubated at 37 °C in a 5.0% CO_2_ incubator at 95% (*v*/*v*) relative humidity (RH %). Cells were detached using 0.25% (*v*/*v*) trypsin containing 1 mM ethylenediaminetetraacetic acid (trypsin-EDTA; Lonza). The phosphate-buffered saline (PBS) used for cell washing and flow cytometry analysis contained 137 mM NaCl, 2.7 mM KCl, 10 mM Na_2_HPO_4_, and 1.8 mM KH_2_PO_4_ (pH 7.4).

To culture and treat cells under physioxia, two different pieces of equipment were used. First, all treatments and cellular manipulations (e.g., passaging and lysis) under physioxia were performed in a physioxia cell culture cabinet (Concept 400, Baker Ruskinn, Sanford, ME, USA) set at 3.0% O_2_/5.0% CO_2_ and 37 °C. Secondly, to grow cells long-term under physioxia, ‘Klip-lock’ airtight containers (Sainsbury’s, London, UK) made from polypropylene (bisphenol A-free) were used (Figure S1a). Each container had a volume of 7.0 L, and was modified with inlet and outlet pipelines, including their associated midline valves. A gas mixture of 3.0% O_2_, 5.0% CO_2_, and 93.0% N_2_ was made up in 10 L, 150 bar cylinders (DDRC Healthcare, Plymouth, UK). A pre-mixed gas cylinder was attached to the inlet valve of the container. As gas flowed through the container, the [O_2_] decreased from 20.9% O_2_ to 3.0% O_2_ within about 2 min (Figure S1b). Closure of both midline valves, upon stabilisation of the [O_2_] set point, produced a gas-tight environment. The container was then placed in a regular cell culture incubator set at 37 °C. To achieve a 95% relative humidity environment during the cell culture stage, a 60 mm Petri dish filled half-way with autoclaved water was also placed into the container with the culture vessels. To maintain airtightness, the rubberised seals on the lid of the container were greased once every fortnight with an inert silicon grease (MolyKote 1102 grease; Dow Corning, Barry, UK). All experimental reagents used for the analysis of cells pre-adapted to physioxia were equilibrated to 3.0% O_2_ by storing these reagents in 3.0% O_2_-gassed airtight plastic containers (Figure S1a) overnight (16 h) at 4 °C.

As such, it was possible to experimentally manipulate the cells whilst maintaining a pre-defined [O_2_] level throughout the course of the experiment: the cells were seeded, underwent changes of medium, or were treated within the physioxia cabinet. The flasks of cultured cells were then placed into the airtight plastic containers within the physioxia cabinet, and moved to a regular cell culture incubator for long-term cell culture growth. The valves on the airtight containers allowed the boxes to be re-gassed at 24 h intervals. Together, this allowed cells to be grown, treated, and prepared for analysis under physioxia without re-oxygenating the cells in room air. In this report, the term “pre-adapted” is used within the context of the [O_2_] utilised during the cell culture growth phase. This term is used to clarify that cells were pre-adapted to either 18.6% or 3.0% O_2_ for their respective time periods (i.e., 24–96 h) prior to treatment with H_2_O_2_, MSA, or CmOOH.

Typically, in each experiment, two 24-well plates were seeded at a density of 9.5 × 10^3^ cells per cm^2^. A431 cells used in these experiments were between passages 5 and 10. Comparisons between cells grown in either [O_2_] (i.e., comparing cells grown in 18.6% O_2_ for 24–96 h to cells grown in 3.0% O_2_ for 24–96 h) were performed on cells derived from the same T75 cm^2^ flask of cells, which was then split over two 24-well plates. A431 cells were pre-adapted to 3.0% or 18.6% O_2_ for (i) 24 h, (ii) 48 h, (iii) 72 h, or (iv) 96 h. The total culture period, regardless of the period of pre-adaptation to 3.0% or 18.6% O_2_, was 96 h. The cells were not passaged over the cell culture growth period whilst growing on the 24-well plate. In each case, the growth medium was replaced with fresh [O_2_]-equilibrated growth medium (either 18.6% or 3.0%) every 24 h. To determine whether the pre-adaptation to 18.6% or 3.0% O_2_ affected the total number of cells in each well prior to further experimentation, cell counts were performed. There was no difference in the total cell count when comparing cells pre-adapted to 18.6% O_2_ to cells pre-adapted to 3.0% O_2_ for 96 h, with cell counts of 6.64 × 10^5^ ± 0.61 vs. 6.50 × 10^5^ ± 0.43, respectively. At the end of cellular pre-adaptation to 18.6% or 3.0% O_2_ for 24–96 h, the following endpoints were analysed: cell death, HIF-1α protein level, lipid peroxidation, antioxidant enzyme activity, catalase protein expression, and Nrf-2 protein expression.

### 2.2. Measurement of the Oxygen Concentration in Experimental Reagents

A blood gas analyser (ABL9; Radiometer Ireland Limited, Lismeehan, Ireland) was used to measure the [O_2_] in all experimental reagents (i.e., culture medium, PBS, etc.) prior to use. Gas-tight vials with rubberised septa (Labco, Lampeter, UK) were utilised to collect the samples within the physioxia cabinet prior to measurement. The [O_2_] in the cell culture medium was measured at the beginning of culture (0 h), at intervals of 24 h during the culture period, and directly prior to experimentation.

### 2.3. Cell Death Analysis by Flow Cytometry

**Figure 1:**
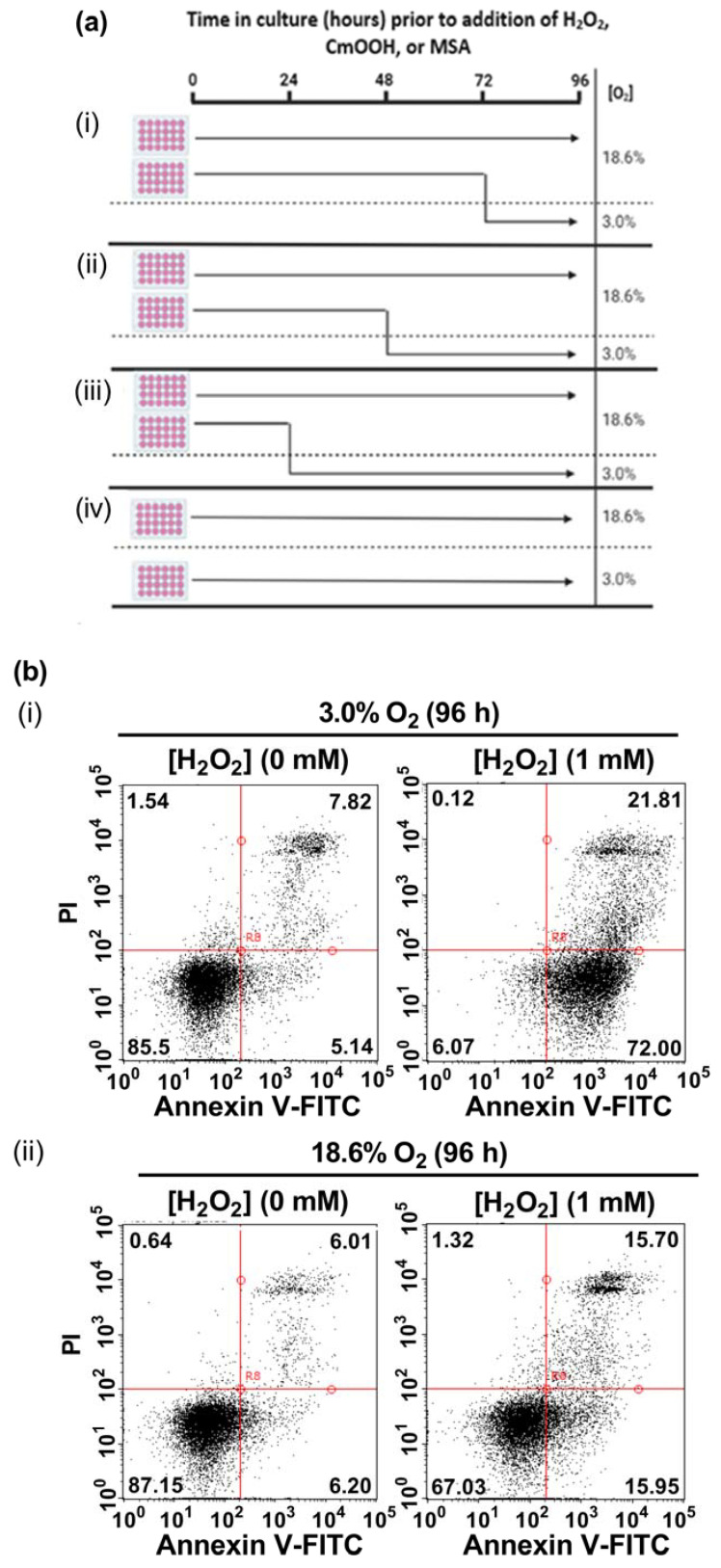

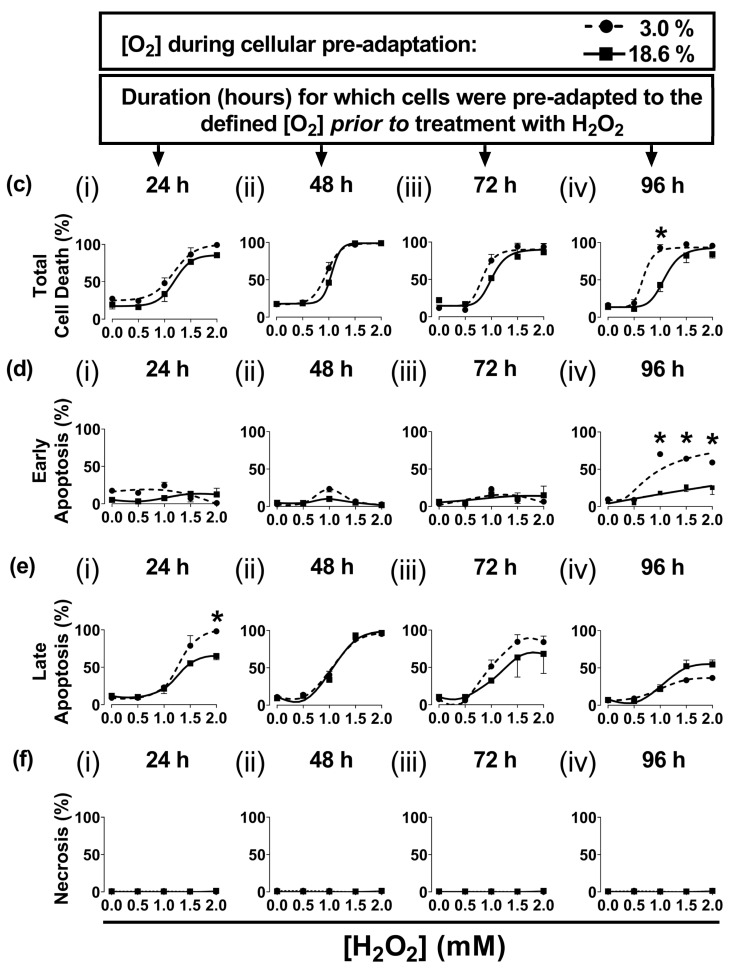
The effect of growing A431 cells in 18.6% O_2_ on cell death induced by H_2_O_2_ compared to cells pre-adapted to 3.0% O_2_. Panel (**a**), a schematic of the experimental design devised for pre-adapting cells to 3.0% or 18.6% O_2_ for (**i**) 24, (**ii**) 48, (**iii**) 72, or (**iv**) 96 h (see Section 2.1 for a detailed description of the experimental design). Panel (**b**), representative dot plot histograms from one experiment indicating cell death in cells pre-adapted to 18.6% or 3.0% O_2_ for 96 h prior to a 1 h treatment with either H_2_O_2_ (1 mM) or 0.1% dH_2_O (vehicle control), as measured by flow cytometry (Section 2.3). The quadrants (vertical and horizontal red lines), and their associated stages of cell death, are defined in Section 2.3. Panel (**c**), graphs showing calculated mean averages for total cell death; panel (**d**), early apoptosis; panel (**e**), late apoptosis; and panel (**f**), necrosis, as measured in cells which were pre-adapted to 18.6% or 3.0% O_2_ for 24 h–96 h and then exposed to varying concentrations of H_2_O_2_ (0–2.0 mM) for 1 h. * = *p* < 0.0001 vs. 3.0% O_2_, utilising a two-way ANOVA and a post hoc multiple comparison test with Dunn–Šidák correction. Data are presented as the mean ± SD; *n* = 3. Where error bars are not visible, this is because the error bar is smaller than the size of the data point. In panel (**e**), the data points represented by the solid circles (3.0% O_2_) are not visible because the data points lie on top of the data points represented by the solid squares (18.6% O_2_). DMSO: dimethyl sulfoxide; FITC: fluorescein isothiocyanate; PI: propidium iodide.

H_2_O_2_ was used to induce cell death. Cells pre-adapted to 3.0% or 18.6% O_2_ for 24–96 h were treated with 0.5–2.0 mM H_2_O_2_, or 0.1% *v/v* dH_2_O alone (vehicle control), for 1 h in 3.0% or 18.6% O_2_ (37.0 °C/5.0% CO_2_). After the treatment period, cells were detached using 0.25% (*v*/*v*) trypsin-EDTA and washed with PBS. Cell death was measured, using annexin V-FITC (#640905, BioLegend, London, UK) and propidium iodide (PI; Lonza) staining, by flow cytometry, using a modified method as described previously [28]. Single staining for annexin V-FITC indicated a cell was in early apoptosis (Figure 1b, bottom right quadrant). Single staining for PI indicated that a cell had undergone necrosis (Figure 1b, top left quadrant). Dual staining for both PI and annexin V-FITC indicated a cell was in late apoptosis (Figure 1b, top right quadrant). Dual negative staining for both annexin V-FITC and PI indicated non-apoptotic cells (Figure 1b, bottom left quadrant). In brief, washed cells were re-suspended in 100 µL of ice cold Ca^2+^ buffer (10 mM HEPES/NaOH, pH 7.4, 140 mM NaCl, 2.5 mM CaCl_2_) containing 1.25 µg/mL annexin V-FITC (BioLegend). After 15 min on ice, and in the dark, 900 µL of Ca^2+^ buffer containing 0.04 mg/mL propidium iodide was added to the cell suspension and the cells were analysed by flow cytometry (Guava EasyCyte^TM^ flow cytometer; Luminex, TX, USA) at excitation 488 nm, with emitted light detected using an FL1 detector (bandpass filter 530 ± 15 nm, abbreviated as 520/30 nm) and an FL3 detector (bandpass filter 695 ± 25 nm, abbreviated as 695/50 nm).

### 2.4. Detection of Lipid Peroxidation

Cells were grown as outlined in Section 2.1. After 96 h of pre-adaptation to 18.6% or 3.0% O_2_, the cells were treated with H_2_O_2_ or CmOOH to induce lipid peroxidation. For testing H_2_O_2_-induced lipid peroxidation, cells which had been pre-adapted to 3.0% O_2_ or 18.6% O_2_ for 96 h were treated with 0.5–2.0 mM H_2_O_2_, or vehicle control alone (0.1% *v/v* dH_2_O), for 1 h in 18.6% or 3.0% O_2_ (37.0 °C/5.0% CO_2_). For testing CmOOH-induced lipid peroxidation, cells which had been pre-adapted to 3.0% O_2_ or 18.6% O_2_ for 96 h were treated with 6.0–200 µM CmOOH, or vehicle control alone (0.1% *v/v* DMSO), for 1 h in 18.6% or 3.0% O_2_ (37.0 °C/5.0% CO_2_). MSA was used to sensitise cells to lipid peroxidation induced by CmOOH as it inhibits GPx [27]. To this end, cells pre-adapted to 18.6% O_2_ or 3.0% O_2_ for 96 h were pre-treated with 0–1000 µM MSA for 24 h in 18.6% or 3.0% O_2_ (37.0 °C/5.0% CO_2_). These cells were then treated with 12.5 µM CmOOH in 18.6% or 3.0% O_2_ (37.0 °C/5.0% CO_2_) for an additional 1 h (37.0 °C/5.0% CO_2_). Lipid peroxidation was then measured as described below.

The cells were detached using 0.25% (*v*/*v*) trypsin-EDTA, washed with PBS, and re-suspended in 1.0 mL of PBS. Under low light, washed cells were re-suspended in 1.0 mL of warm PBS containing 1 µM of 4,4-difluoro-5-(4-phenyl-1,3-butadienyl)-4-bora-3a,4a-diaza-s-indacene-3-undecanoic acid (C11-BODIPY^581/591^; Thermo Fisher), using a modified method as described previously [29]. Lipid peroxidation was detected by flow cytometry at excitation 488 nm, with the emitted light of oxidised C11-BODIPY^581/591^ (O.BOD (+)) detected using the FL1 detector (green) and the emitted light of non-oxidised C11-BODIPY^581/591^ (R.BOD (+)) detected using the FL2 detector (red). Data for the C11-BODIPY^581/591^ studies are presented as a ratio of the green fluorescent signal to the red fluorescent signal, as detected by the FL1 and FL2 (bandpass filter 583 ± 13 nm, abbreviated as 583/26) detectors, respectively. This is abbreviated as F520:F583.

### 2.5. Measurement of H_2_O_2_ by Amplex Red

For the measurements of H_2_O_2_ concentrations, the fluorescent probe Amplex Red (#A12222; Thermo Fisher) was utilised according to the manufacturer’s instructions. In brief, cells were seeded at a density of 9.5 × 10^3^ cells per cm^2^ in black 96-well plates and were then pre-adapted to 18.6% O_2_ or 3.0% O_2_ for 96 h (37 °C/5.0% CO_2_). Fresh appropriate [O_2_]-equilibrated growth medium (18.6% or 3.0%) was used to replace the spent medium above the cells every 24 h. After 96 h of growth, an appropriate [O_2_]-equilibrated (18.6% or 3.0% O_2_) Amplex Red reaction mixture (Amplex Red (100 µM) and HRP (0.2 U/mL)) was added to the appropriate wells under low light (1:1 dilution). Both the 3.0% and 18.6% O_2_ plates were sealed with gas-impermeable cellophane tape (Tuff Tape, StormSure Ltd., Cambridge, UK) to maintain the gas environment of both conditions during analysis. The fluorescence of the resorufin end product was measured at excitation 570 nm and emission 585 nm (bottom read) at intervals of 30 min for 2 h utilising a Spectramax M2e spectrophotometer. [H_2_O_2_] was determined by interpolation from an Amplex Red standard curve. After 96 h of cell culture and an Amplex Red-based detection of H_2_O_2_, the cell number was determined using an automatic cell counter (TC20; Bio-Rad).

### 2.6. Whole Cell and Nuclear Lysis

Cellular lysates were prepared prior to carrying out antioxidant enzyme activity assays or Western blotting. Cells were cultured for the desired length of time in 3.0% O_2_ or 18.6% O_2_ in T75 flasks (Greiner, Stonehouse, UK).

For whole cell lysis, whole cell lysis solution was prepared by adding one protease inhibitor tablet (#A32963; Thermo Fisher, UK) to 10 mL of radio immunoprecipitation assay (RIPA) buffer (10 mM Tris-HCl, 1 mM EDTA, 0.5 mM EGTA, 1 mM PMSF, 140 mM NaCl, 1% *v/v* Triton X-100, 0.1% *v/v* sodium deoxycholate, and 0.1% *v/v* SDS, pH 8.0). The cells were washed twice with an appropriate (18.6% or 3.0%) [O_2_]-equilibrated PBS prior to adding 1 mL of whole cell lysis solution. The flasks were kept on ice for 5 min and the cell layer was then scraped using a cell scraper (Thermo Fisher). The crude mixture was transferred to a new tube and centrifuged at 10,000× *g* for 15 min at 4 °C. The supernatant of the resulting solution was stored on ice or frozen (−80 °C) until use. Its protein concentration was measured using a PierceTM bicinchoninic acid (BCA) protein assay kit (Thermo Fisher).

For nuclear lysis, the method of Schreiber et al. [30] was utilised. Nuclear extracts were used for semi-quantifying the levels of HIF-1α and Nrf-2 by Western blotting. The cells were washed with appropriate (18.6% or 3.0%) [O_2_]-equilibrated PBS, detached with 0.25% *v/v* trypsin-EDTA, and pelleted by centrifugation at 200× *g* for 2 min. The cells were then re-suspended in ice-cold cell lysis buffer (10 mM HEPES, 10 mM KCl, 0.1 mM EDTA, 1 mM dithiothreitol (DTT), 0.5% Nonidet-40, 0.5 mM PMSF, pH 7.5) with a protease inhibitor cocktail (containing 10 µM leupeptin, 10 µM E-64, 10 µM bestatin, 0.3 µM aprotinin, and 0.5 mM 4-(2-aminoethyl) benzenesulfonyl fluoride hydrochloride; #ab65621; Abcam, Cambridge, UK). The cells were left to swell on ice for 15 min with intermittent mixing every 3 min using a vortex mixer set at 200 rpm. The sample was then centrifuged at 12,000× *g* at 4 °C for 1 min. The pellet was then re-suspended in nuclear extraction buffer (20 mM HEPES, 400 mM NaCl, 1 mM EDTA, 1 mM DTT, 1 mM PMSF, pH 7.5) with a protease inhibitor cocktail. The pellet was then incubated on ice for 30 min with intermittent mixing at intervals of 5 min using a vortex mixer (200 rpm). This lysate was then pelleted by centrifugation at 14,000× *g* for 15 min at 4 °C. The protein concentration in the nuclear extract was estimated using the Pierce^TM^ BCA protein assay kit.

### 2.7. Catalase Activity

Cells were seeded at a density of 9.5 × 10^3^ cells per cm^2^ in a six-well plate and were pre-adapted to 18.6% O_2_ or 3.0% O_2_ for 24–96 h (37.0 °C/5.0% CO_2_), as described in Section 2.1. Cells were subjected to whole-cell lysis, as described below (Section 2.11). Catalase (CAT) activity was then measured, using the method described by Li and Schellhorn [31], by monitoring the decomposition of H_2_O_2_ via spectrophotometry. Briefly, in a quartz cuvette, 100 µg of sample protein was added to 5 mM H_2_O_2_ dissolved in 50 mM phosphate buffer (pH 7.4). The change in absorbance at 240 nm (ΔA_240_) was immediately monitored at intervals of 5 s for 2 min at 22 °C using a Spectramax M_2_e spectrophotometer (Molecular Devices). Known concentrations of bovine CAT (Merck, 0–200 U) were also assayed, allowing for interpolation of the sample’s enzyme activity from a standard curve, where 1/slope of the initial linear reaction (ΔA_240_ 0–1 min) was plotted against [CAT]. One unit of CAT decomposes 1 μmol of H_2_O_2_ per min at pH 7.4 at 25 °C.

### 2.8. Superoxide Dismutase Activity

Cells were grown as described in Section 2.7. Superoxide dismutase (SOD) activity was measured in cell lysates, using the method described previously by Peskin and Winterbourn et al. [32], by monitoring the hypoxanthine and xanthine oxidase-generated superoxide-dependent reduction of tetrazolium dye 2-(4-iodophenyl)-3-(4-nitrophenyl)-5-(2,4-disulfophenyl)-2H-tetrazolium sodium salt (WST1; Merck). In brief, assay buffer (50 mM sodium phosphate, 0.1 mM DTPA, 0.1 mM hypoxanthine, 50 µM WST-1, pH 7.8) was added to a 96-well plate containing 100 µg of sample protein. The reaction was initiated by adding xanthine oxidase (final activity 10 mU), and the plate was shaken vigorously for 30 s. WST-1 reduction was measured by monitoring ΔA_438_ at 25 °C at intervals of 10 s for 2 min using a Spectramax M_2_e spectrophotometer. Known concentrations of bovine SOD (Merck, 0–40 U/mL) were also assayed, allowing for interpolation of the sample’s enzyme activity from a standard curve, where 1/slope of the initial linear reaction (ΔA_438_ 0–1 min) was plotted against [SOD]. One unit of SOD inhibits WST-1 reduction by 50% in a coupled system with xanthine oxidase at pH 7.8 at 25 °C.

### 2.9. Glutathione Reductase Activity

Cells were grown as described in Section 2.7. Glutathione reductase (GR) activity was measured in cell lysates, using a modified version of the method described by Mannervik et al. [33], by monitoring the NADPH-dependent reduction of oxidised glutathione (GSSG). In brief, assay buffer (200 mM potassium phosphate, 2 mM EDTA, 1 mM GSSG, 200 µM NADPH, pH 7.6) was added to a 96-well plate containing 100 µg of sample protein. The plate was shaken vigorously for 30 s and the oxidation of NADPH was measured by monitoring ΔA_340_ at 25 °C at intervals of 10 s for 2 min using a Spectramax M_2_e spectrophotometer. Known concentrations of GR from baker’s yeast (0–40.0 U/mL; Merck) were also assayed, allowing for interpolation of the samples against a standard curve, where 1/slope of the initial linear reaction (ΔA_340_ 0–1 min) was plotted against [GR]. One unit of GR reduces 1 μmol of oxidised glutathione per min at pH 7.6 at 25 °C.

### 2.10. Glutathione Peroxidase Activity

Cells were grown as described in Section 2.7. Glutathione peroxidase (GPx) activity was measured in cell lysates, using a modified version of the method described by Mannervik et al. [33], by monitoring the NADPH-dependent reduction of GSSG. In brief, assay buffer (50 mM Tris-HCl, 5 mM EDTA, 1 mg/mL BSA, pH 7.4) was added to a 96-well plate containing 100 µg of sample protein. A co-substrate mixture containing 1 mM GSH and 1 mU GR (dissolved in assay buffer) was added to equal volumes of GPx sample in a clear-bottom 96-well plate. NADPH (Merck), made up in assay buffer, was then added to a final concentration of 240 µM. The reaction was initiated by adding H_2_O_2_ to a final concentration of 100 µM. The plate was shaken vigorously for 30 s and the oxidation of NADPH was measured by monitoring ΔA_340_ at intervals of 5 s for 1 min using a Spectramax M_2_e spectrophotometer. Known concentrations of GPx (0–1 mU/mL; Merck) were also assayed, allowing for interpolation of sample’s GPx activity from a standard curve, where 1/slope of the initial linear reaction (ΔA_340_ 0–1 min) was plotted against GPx activity. One unit of GPx catalyses the H_2_O_2_-dependent oxidation of 1 μmol of reduced glutathione to oxidised glutathione per min at pH 7.4 at 25 °C.

### 2.11. Detection of Transcription Factors (Hypoxia-Inducible Factor-1α and Nrf-2) and Catalase Protein by Western Blotting

To measure the levels of CAT or Nrf-2 protein, A431 cells were seeded at a density of 9.5 × 10^3^ cells per cm^2^ in a 6-well plate and were then pre-adapted to 18.6% O_2_ or 3.0% O_2_ for 24–96 h (37.0 °C/5.0% CO_2_) as described previously (Section 2.1). The cells then underwent whole-cell lysis for the detection of catalase protein, or nuclear lysis for the detection of Nrf-2 protein, as described in Section 2.6. To measure the levels of HIF-1α, A431 cells were seeded at a density of 9.5 × 10^3^ cells per cm^2^ in a 6-well plate and were then pre-adapted to 18.6% O_2_ or 3.0% O_2_ for 24–96 h (37.0 °C/5.0% CO_2_). As a positive control for HIF-1α protein expression, the cells were pre-adapted to 0.5% O_2_ for 1 h. The cells then underwent nuclear lysis (Section 2.1).

Samples of whole-cell lysate (10 µg), or nuclear lysate (50 µg), were incubated at 95 °C for 5 min in Laemmli buffer containing 50 mM dithiothreitol. The proteins were separated by gel electrophoresis at 100 V for 90 min using pre-cast 8–16% polyacrylamide gels (BioRad, Watford, UK) in sodium dodecyl sulphate (SDS) buffer (30.3 g Tris base, 144.2 g glycine, 10 g SDS made up to 1 L in dH_2_O). Protein transfer was performed utilising a nitrocellulose TransBlot Turbo transfer system kit (BioRad). The membrane was then blocked with protein-free blocking buffer (#37572; Thermo Fisher) containing 0.1% (*v/v*) Tween-20 (TPBS) overnight (16 h) at 4 °C. Each respective membrane was then incubated for 1 h at room temperature with the appropriate primary antibody targeting HIF-1α (mouse; #NB100-105); CAT (mouse; #NBP2-00492), from Bio-Techne, Abingdon, UK; or Nrf-2 (mouse; #12721) from Cell Signalling, London, UK. The primary antibodies were diluted 1:1000 in PBS. Staining for GAPDH (rabbit; #NB300-322; Bio-Techne), or total protein (Revert™ Total Protein Stain kit; LICOR, Cambridge, UK), was used to check lane-to-lane variations in protein loading to control for loading error in the CAT Western blotting experiments or the HIF-1α and Nrf-2 Western blotting experiments, respectively. Total protein stains were then imaged using an Azure 600 Western blotting imaging system (Azure Biosystems, CA, USA). The membranes were then washed 5 times with TPBS (at intervals of 1 min) and incubated for 1 h at room temperature with IRDye 800CW goat anti-mouse polyclonal (1:10,000; #926-32210) or IRDye 680 goat anti-rabbit polyclonal secondary antibody (1:10,000; #926-68071) from LI-COR. The membrane was then washed three times with TPBS at intervals of 1 min and was imaged using a LI-COR Odyssey CLx imaging system. Densitometry analysis was performed using Image Studio^TM^ Lite quantitation software v5.0 (LI-COR Biosciences UK Ltd., Cambridge, UK).

### 2.12. Statistical Analysis

Data are presented as the mean ± one standard deviation of the indicated number of independent experiments. Where error bars are not visible, this is because the error bar is smaller than the size of the data point. Statistical significance was calculated using a two-way ANOVA and a post hoc multiple comparison test. *p* ≤ 0.05 was considered statistically significant.

## 3. Results

### 3.1. Cells Pre-Adapted to 18.6% O_2_ Are Resistant to H_2_O_2_-Induced Cell Death Compared to Cells Pre-Adapted to 3.0% O_2_

In the present study, A431 cells were pre-adapted to either 18.6% O_2_ or 3.0% O_2_ for 24–96 h, before being exposed to H_2_O_2_ (see the schematic of the experimental design in Figure 1a). A representative dot plot histogram (Figure 1b) from the 96 h time point in one experiment shows the viable cells (bottom left-hand quadrant), early apoptotic cells (bottom right-hand quadrant), late apoptotic cells (top right-hand quadrant), and necrotic cells (top left-hand quadrant). The results from the raw data dot plots were analysed to generate the cell death response curves, following exposure to increasing concentrations of H_2_O_2,_ shown in Figure 1c–f, as obtained for the O_2_ pre-adaptation periods of 24, 48, 72, and 96 h.

Cells pre-adapted to 18.6% O_2_ for 96 h, and subsequently treated with 1.0 mM H_2_O_2_, showed decreased total cell death compared to cells pre-adapted to 3.0% O_2_ for 96 h (*p* < 0.0001, Figure 1c(iv)), with 43.0 ± 7.1% cell death vs. 92.9 ± 3.6% cell death, respectively. This difference in total cell death was largely due to the differences in the percentage of cells in early apoptosis (Figure 1d(iv)). Thus, cells pre-adapted to 18.6% O_2,_ and subsequently treated with 1.0 mM H_2_O_2_ (as well as the cells treated with 1.5 and 2.0 mM H_2_O_2_), showed a decrease in their percentage of early apoptotic cells compared to cells adapted to 3.0% O_2_ for 96 h (*p* < 0.0001, Figure 1d(iv)). When comparing the cells which had been pre-adapted to 18.6% O_2_ vs. cells pre-adapted to 3.0% O_2_, respectively, the percentages of early apoptotic cells were: 18.0 ± 1.6% vs. 70.2 ± 3.0% in early apoptosis after the 1.0 mM H_2_O_2_ treatment, 26.5 ± 6.5% vs. 64.2 ± 3.4% after the 1.5 mM H_2_O_2_ treatment, and 25.3 ± 9.2% vs. 59.0 ± 0.5% after the 2.0 mM H_2_O_2_ treatment. Whilst cells pre-adapted to 3.0% for 24 h showed an increase in their percentage of cells in late apoptosis when then treated with 2.0 mM H_2_O_2_ compared to cells pre-adapted to 18.6% O_2_ (Figure 1e(i)), this difference was not seen when comparing the other pre-adaptation time-points (Figure 1e(ii–iv)). There was no difference in the percentage of cells in necrosis (Figure 1f) when comparing cells pre-adapted to either 18.6% or 3.0% O_2_ for 24–96 h which were then treated with 0.5–2.0 mM H_2_O_2_.

**Figure 2:**
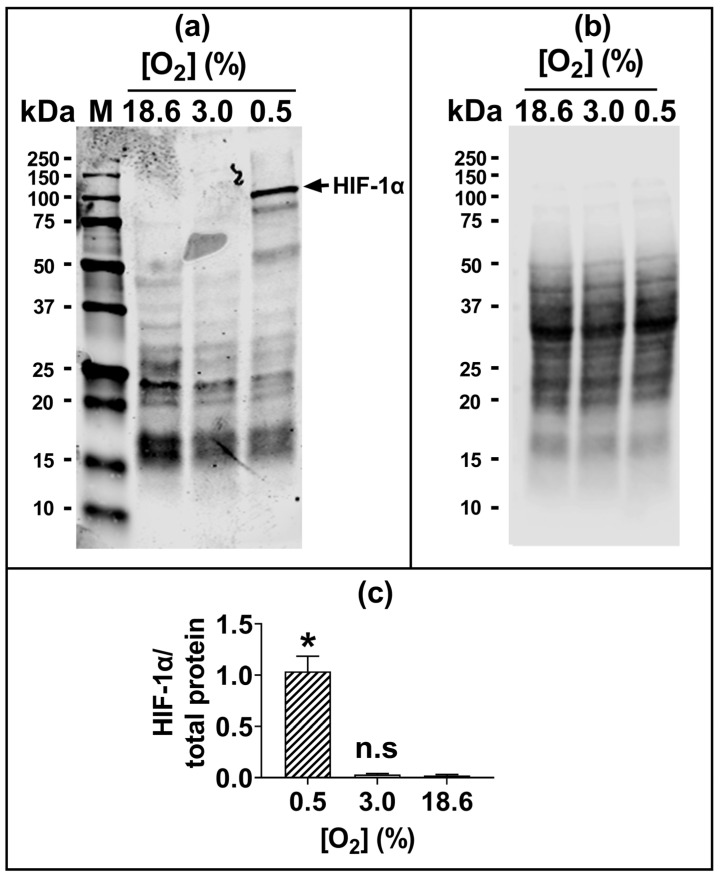
The expression of HIF-1α protein in A431 cells grown in 3.0% O_2_ or 18.6% O_2_ for 96 h. A431 cells were pre-adapted to 18.6% O_2_ or 3.0% O_2_ for 96 h (for methods, see Section 2.11). As a positive control for HIF-1α protein expression, A431 cells were exposed to 0.5% O_2_ for 1 h (Section 2.11). Panel (**a**), representative full-length immunoblot for HIF-1α protein expression (band of interest, 93 kDa, HIF-1α). Densitometry analysis was performed on the 93 kDa band and was normalised to the total protein (Section 2.11). The immunoblot bands with molecular weights less than 93 kDa may represent degradation breakdown products of HIF-1α. Panel (**b**), total protein staining, corresponding to the blot shown in panel (**a**), used for normalisation purposes (imaged using an Azure Biosystems Western blotting imaging system). Panel (**c**), HIF-1α nuclear protein levels in cells pre-adapted to 18.6% or 3.0% O_2_ relative to the levels in cells pre-adapted to 0.5% O_2_ for 1 h. The data values in panel (c) are presented as the mean ± SD; *n* = 3. n.s = *p* > 0.05 versus 18.6% O_2_, * = *p* < 0.0001 versus 3.0% O_2_ using a two-tailed Student’s *t*-test. HIF: hypoxia-inducible factor; kDa: kilodalton; M: molecular weight marker lane.

The 96 h pre-adaptation time point was chosen to test further end points (e.g., HIF1-α and lipid peroxidation), as this time point showed the largest [O_2_]-dependent difference in H_2_O_2_-induced cell death (Figure 1c(iv); Figure 1d(iv)). There was no statistically significant difference in the nuclear protein levels of HIF1-α when comparing cells pre-adapted to 18.6 O_2_ for 96 h to cells pre-adapted to 3.0% O_2_ for 96 h (Figure 2).

### 3.2. Cells Pre-Adapted to 18.6% O_2_ Are Resistant to H_2_O_2_- and Cumene Hydroperoxide-Induced Lipid Peroxidation Compared to Cells Pre-Adapted to 3.0% O_2_

**Figure 3:**
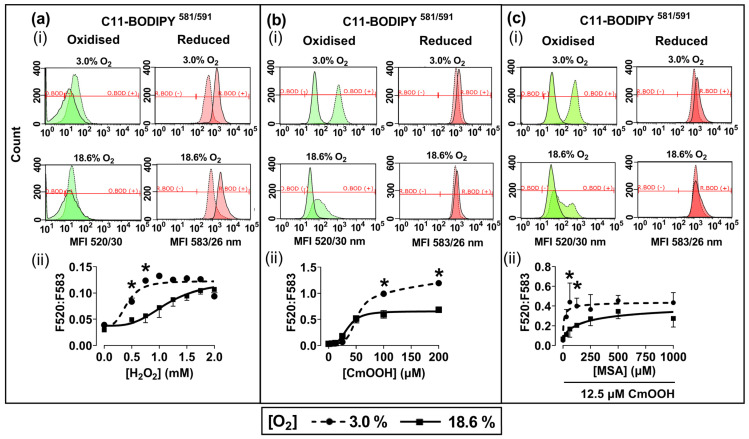
The effect of growing A431 cells in 18.6% O_2_ or 3.0% O_2_ on their subsequent lipid peroxidation induced by H_2_O_2_, or by cumene hydroperoxide, in the presence or absence of mercaptosuccinic acid. A431 cells were pre-adapted to 18.6% or 3.0% O_2_ for 96 h (for methods, see Section 2.1) and subsequently treated with H_2_O_2_, CmOOH, or MSA, prior to measuring lipid peroxidation (Section 2.4). Panel (**a**): (**i**) representative fluorescence histogram showing lipid peroxidation in cells treated with H_2_O_2_ (0.75 mM) for 1 h. Solid curves indicate untreated cells and dashed curves indicate treated cells; (**ii**) lipid peroxidation in cells pre-adapted to 18.6% O_2_ or 3.0% O_2_ for 96 h and then treated with 0.0–2.0 mM H_2_O_2_ for 1 h. Panel (**b**): (**i**) representative fluorescence histogram showing lipid peroxidation in cells treated with CmOOH (100 µM) for 1 h; (**ii**) lipid peroxidation in cells pre-adapted to 18.6% O_2_ or 3.0% O_2_ for 96 h and then treated with 0–200 µM CmOOH for 1 h. Panel (**c**): (**i**) representative fluorescence histogram showing lipid peroxidation in cells pre-treated with MSA (125 µM) for 24 h prior to exposure to 12.5 µM CmOOH for 1 h; (**ii**) lipid peroxidation in cells pre-adapted to 18.6% O_2_ or 3.0% O_2_ for 96 h which were then pre-treated with 0–1000 µM MSA for 24 h prior to exposure to 12.5 µM CmOOH for 1 h. * = *p* < 0.0001 versus 18.6% O_2_, utilising a two-way ANOVA and a post hoc multiple comparison test with Dunn–Šidák correction. Data are presented as the mean ± SD; *n* = 4. CmOOH: cumene hydroperoxide; F520:F583: mean fluorescence intensity ratio of the light emission at 520 nm to the light emission at 583 nm, when excited at 488 nm; MSA: mercaptosuccinic acid. O.BOD (−/+): oxidised C11-BODIPY^581/591^ negative or positive regions; R.BOD (−/+): reduced C11-BODIPY^581/591^ negative or positive regions.

Representative fluorescence histograms (Figure 3a–c(i)) indicate the MFI of oxidised C11-BODIPY^581/591^ (green histogram) or reduced C11-BODIPY^581/591^ (red histogram) after treating the pre-adapted cells with H_2_O_2_ or CmOOH (dashed traces), compared to untreated controls (solid traces). The MFIs from these histograms were used to calculate the fluorescence ratios (F520:583) that are indicative of these lipid peroxidation levels, as described previously in Section 2.4. Cells pre-adapted to 18.6% O_2_ for 96 h, and subsequently treated with 0.75 mM H_2_O_2_, showed a decrease in lipid peroxidation compared to cells pre-adapted to 3.0% O_2_ for 96 h (*p* < 0.0001, Figure 3a(ii)), with F520:583 values of 0.73 ± 0.12 vs. 1.23 ± 0.08, respectively.

Cells pre-adapted to 18.6% O_2_ for 96 h, and then treated with 200.0 µM CmOOH, showed a decrease in CmOOH-induced lipid peroxidation compared to cells pre-adapted to 3.0% O_2_ for 96 h (*p* < 0.0001; Figure 3b(ii)), with F520:583 values of 0.68 ± 0.02 vs. 1.19 ± 0.03, respectively. Cells pre-adapted to 18.6% O_2_ for 96 h, pre-treated with 60.0 µM of the GPx inhibitor MSA, and then treated with 12.5 µM CmOOH, showed a decrease in lipid peroxidation compared to cells pre-adapted to 3.0% O_2_ for 96 h (*p* < 0.0001, Figure 3c(ii)), with F520:583 values of 0.17 ± 0.07 vs. 0.44 ± 0.17.

### 3.3. Cells Pre-Adapted to 18.6% O_2_ Exhibit Higher Antioxidant Enzymatic Activities of Catalase and Glutathione Reductase Compared to Cells Pre-Adapted to 3.0% O_2_

**Figure 4:**
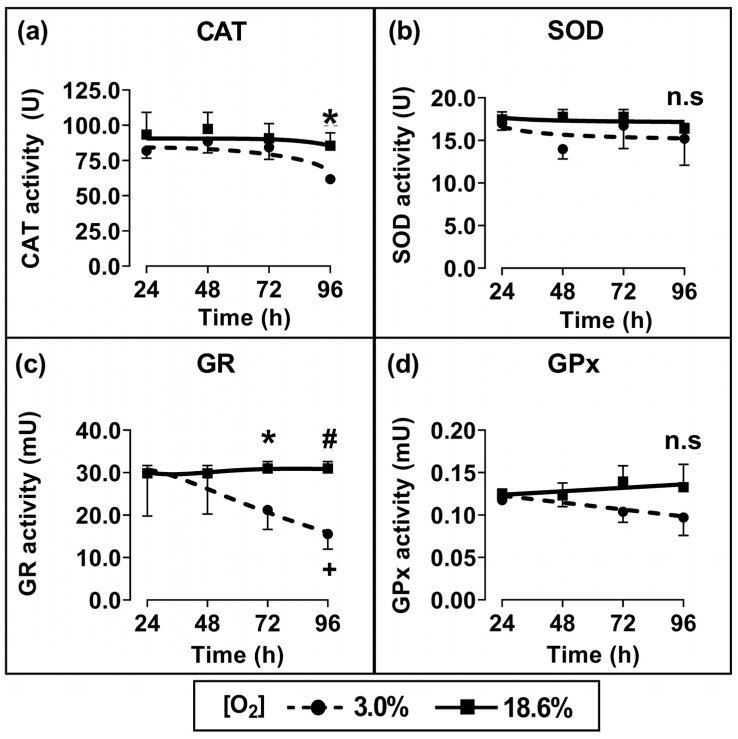
The effect of growing A431 cells in 18.6% O_2_ on the activities of antioxidant enzymes compared to cells pre-adapted to 3.0% O_2_. Panels (**a**–**d**): the enzymatic activities of (**a**) catalase (for methods, see Section 2.7), (**b**) SOD (Section 2.8), (**c**) GR (Section 2.9), or (**d**) GPx (Section 2.10), measured in the whole-cell lysate of cells pre-adapted to 18.6% or 3.0% O_2_ for 24–96 h (Section 2.1). The enzymatic activities were normalised to the ‘activity per mg of sample protein’. n.s = not significant, * = *p* < 0.05, # = *p* < 0.01 versus 3.0% O_2_, + = *p* < 0.05 versus 24 h 3.0% O_2_, utilising a two-way ANOVA and a post hoc multiple comparison test with Tukey correction. Data are presented as the mean ± SD; *n* = 4. Where error bars are not visible this is because the error bar is smaller than the size of the data point. CAT: catalase; GPx: glutathione peroxidase; GR: glutathione reductase; SOD: superoxide dismutase.

Cells which had been pre-adapted to 18.6% for 96 h showed a statistically significant increase in CAT activity (*p* < 0.05; Figure 4a) and GR activity (*p* < 0.01, Figure 4c) compared to cells pre-adapted to 3.0% O_2_ for 96 h. When comparing cells which had been pre-adapted to 18.6% O_2_ with cells which had been pre-adapted to 3.0% O_2_, their respective measured mean (±SD) enzyme activities were 85.5 U ± 7.1 vs. 61.8 U ± 2.7 for CAT and 30.2 ± 1.9 mU vs. 15.5 ± 3.3 mU for glutathione reductase. Such differences were not statistically significant for the shorter pre-adaptation time periods. Additionally, there was no statistically significant difference in the enzymatic activities of SOD (Figure 4b) or GPx (Figure 4d) when comparing cells pre-adapted to 18.6% O_2_ to cells pre-adapted to 3.0% O_2_ for 24–96 h.

### 3.4. Cells Pre-Adapted to 18.6% O_2_ Exhibit a Higher Level of Catalase Protein, and a Higher H_2_O_2_ Concentration, Compared to Cells Pre-Adapted to 3.0% O_2_ for 96 h

**Figure 5:**
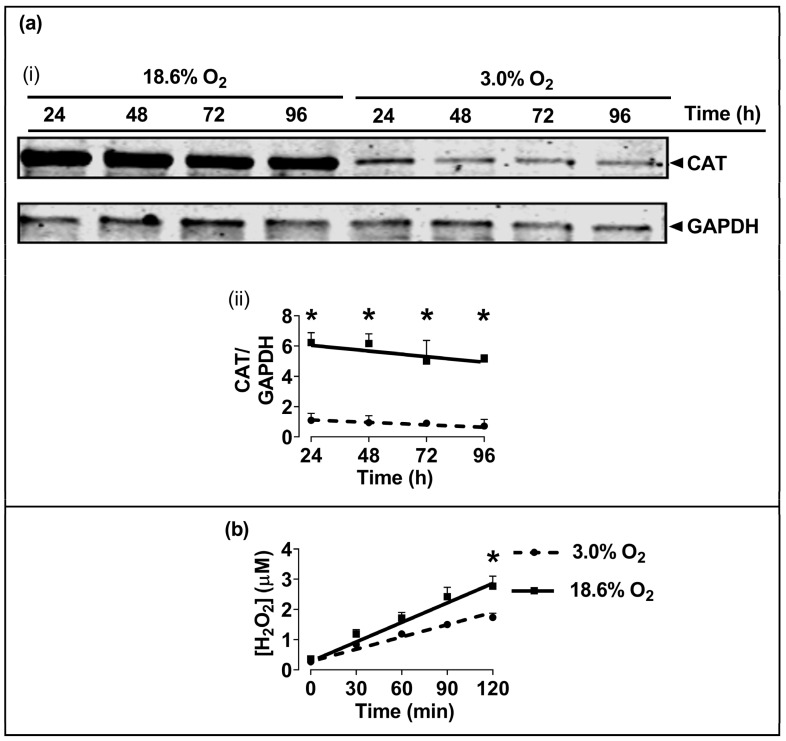
The effect of growing A431 cells in 18.6% O_2_ on the levels of catalase protein, and on the generation of H_2_O_2_, compared to cells pre-adapted to 3.0% O_2_. Panel (**a**), the levels of CAT protein in cells pre-adapted to 18.6% O_2_ or 3.0% O_2_ for 24–96 h (for methods, see Section 2.11), shown by (**i**) a representative immunoblot from one experiment showing the 60 kDa CAT band of interest (the 36 kDa band is GAPDH, which was the loading control), and (**ii**) combined densitometric analysis of the 60 kDa CAT band, denoting the mean average levels of CAT protein expression in cells pre-adapted to 18.6% O_2_ or 3.0% O_2_ relative to the levels in cells pre-adapted to 3.0% O_2_ for 24 h. Panel (**b**), Amplex red-mediated detection (Section 2.5) of extracellular H_2_O_2_ generation in cells pre-adapted to 18.6% or 3.0% O_2_ for 96 h. The graph shows the time-course of the measured H_2_O_2_ concentration in cells pre-adapted to 18.6% O_2_ or 3.0% O_2_ for 96 h, and then maintained at these same O_2_ concentrations for 120 min during fluorescence monitoring after the addition of Amplex Red. * = *p* < 0.001 versus 3.0% O_2_, utilising a two-way ANOVA and a post hoc multiple comparison test with Dunn–Šidák correction. Data are presented as the mean ± SD; *n* = 4. Where error bars are not visible, this is because the error bar is smaller than the size of the data point. CAT: catalase; GAPDH: glyceraldehyde 3-phosphate dehydrogenase; HRP: horseradish peroxidase.

The Western blotting of lysates from cells which had been pre-adapted to 18.6% O_2_ for 96 h showed higher levels of immuno-detected CAT protein compared to cells pre-adapted to 3.0% O_2_ for 96 h (*p* < 0.001, Figure 5a), with normalised CAT band intensities (CAT/GAPDH) of 5.2 ± 0.3 vs. 0.9 ± 0.2 after cellular pre-adaptation to 18.6% and 3.0% O_2_, respectively. Cells pre-adapted to 18.6% O_2_ for 96 h also showed increased basal H_2_O_2_ levels when measured over a 2 h culture period compared to cells pre-adapted to 3.0% O_2_ for 96 h (*p* < 0.001, Figure 5b), with 2.77 ± 0.33 vs. 1.73 ± 0.14 µM H_2_O_2_, respectively. At the end of the Amplex Red incubation, a cell count showed that there were 9.8 × 10^4^ (±0.2 × 10^4^) cells per well in the 18.6% O_2_ group versus 9.2 × 10^4^ (±0.7 × 10^4^) in the 3.0% O_2_ group.

### 3.5. Cells Pre-Adapted to 18.6% O_2_ Exhibit a Higher Level of Nuclear Nrf-2 Protein Compared to Cells Pre-Adapted to 3.0% O_2_ for 96 h

**Figure 6:**
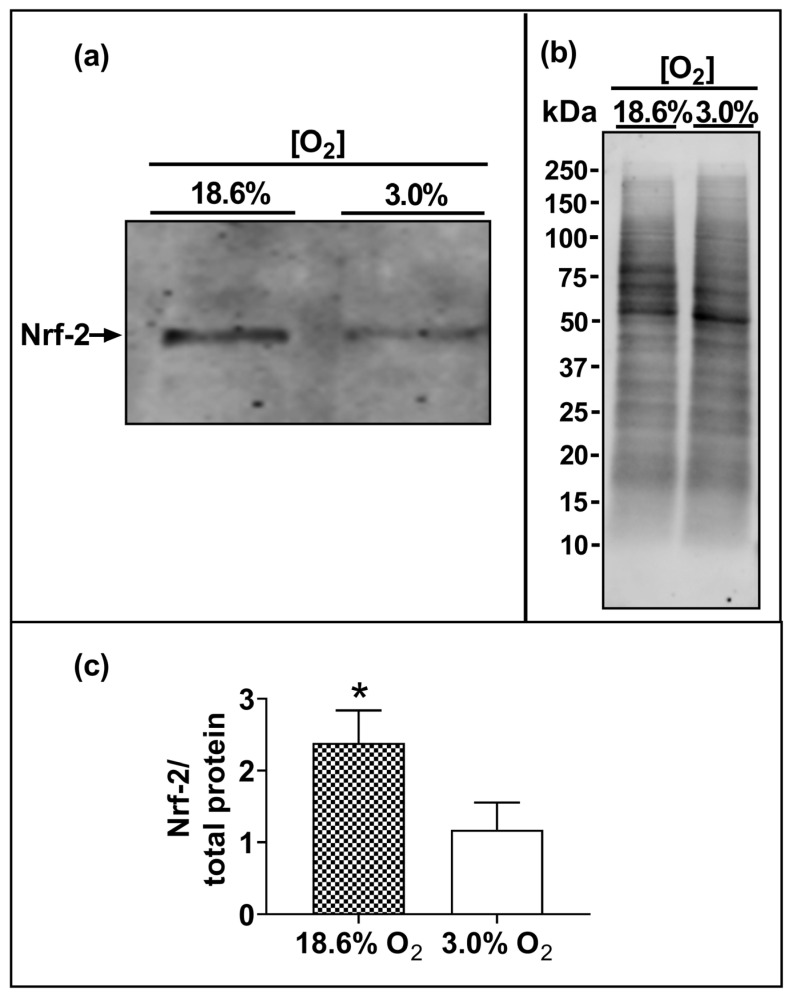
The effect of growing A431 cells in 18.6% O_2_ on the nuclear levels of Nrf-2 protein compared to cells pre-adapted to 3.0% O_2_. Panel (**a**), representative immunoblot from one experiment showing the 95 kDa band of interest, Nrf-2. Densitometry analysis was performed on the 95 kDa band and was normalised to the total protein. Panel (**b**), total protein staining (for methods, see Section 2.11), corresponding to the blot shown in panel (**a**), used for normalisation purposes (imaged using an Azure Biosystems Western blotting imaging system). Panel (**c**), Nrf-2 nuclear protein levels measured in the nuclear lysates (Section 2.11) of cells pre-adapted to 18.6% O_2_ for 96 h, relative to the levels in cells pre-adapted to 3.0% O_2_ for 96 h. The data values in panel (**c**) are presented as the mean ± SD; *n* = 3. * = *p* < 0.05 versus 3.0% O_2_, using a two-tailed Student’s *t*-test. kDa: kilodalton; Nrf-2: nuclear factor erythroid 2-related factor 2.

The Western blotting of lysates from cells which had been pre-adapted to 18.6% O_2_ for 96 h showed higher levels of immuno-detected nuclear Nrf-2 protein compared to cells pre-adapted to 3.0% O_2_ for 96 h (*p* < 0.001, Figure 6c), with normalised Nrf-2 band intensities (Nrf-2/total protein) of 2.4 ± 0.4 vs. 1.2 ± 0.3 after cellular pre-adaptation to 18.6% and 3.0% O_2_, respectively.

## 4. Discussion

The present study showed that human non-melanoma squamous cell carcinoma cells (A431 cells), when pre-adapted to a standard cell culture oxygenation condition (18.6% O_2_) and then exposed to a bolus addition of H_2_O_2_, resisted H_2_O_2_-induced cell death compared to A431 cells pre-adapted to skin physioxia (3.0% O_2_) for 96 h. The sensitisation to H_2_O_2_ treatment of cells pre-adapted to 3.0% O_2_ (Figure 1c(iv)) was not due to the activation of HIF-1α. There was no significant difference in the nuclear protein levels of HIF-1α when comparing cells pre-adapted to 3.0% O_2_ for 96 h to cells pre-adapted to 18.6% O_2_ for 96 h (Figure 2c), which agrees with a previous report from our laboratory [15]. To activate HIF-1α in A431 cells, researchers have previously used an [O_2_] of ≤1.0% O_2_ [34,35,36,37,38,39]. Our results suggest that A431 cells, when pre-adapted to 3.0% O_2_ for 96 h, were sensitised to H_2_O_2_-induced cell death in a manner which was independent of hypoxic conditions and HIF-1α activation.

The resistance to H_2_O_2_-induced cell death of cells which had pre-adapted to 18.6% O_2_ (Figure 1c) was largely due to a decrease in the percentage of cells in early apoptosis (Figure 1d), when compared to cells pre-adapted to 3.0% O_2_ for 96 h. The duration of the pre-adaptation time in 3.0% O_2_, prior to a H_2_O_2_ treatment, was an important experimental parameter. For example, cells pre-adapted to 3.0% O_2_ for just 24 h prior to H_2_O_2_ treatment (Figure 1c(i)) showed no significant difference in total cell death when compared to cells pre-adapted to 18.6% O_2_. This suggests that the phenotype of the cells was changing over the 96 h pre-adaptation period in physioxia (3.0% O_2_), relative to the cells pre-adapted to 18.6% O_2_ for 96 h (Figure 1c,d). The maximum pre-adaptation time period tested in the present study was 96 h. Cells were seeded at a density (9.5 × 10^3^ cells/cm^2^) which allowed the adherent cells to grow during this 96 h period without becoming confluent, thus avoiding a cell passage during the growth of cells for 24–96 h (Figure 1a). The experiment was designed in this way because the passaging of cells could be a potential confounding factor when assessing the effects of cell growth at different [O_2_] concentrations for defined time periods.

Further experiments were performed to determine whether the resistance to H_2_O_2_-induced cell death of cells pre-adapted to 18.6% O_2_ extended to H_2_O_2_-induced lipid peroxidation. It was found that A431 cells pre-adapted to 18.6% O_2_ for 96 h were not only resistant to H_2_O_2_-induced lipid peroxidation (Figure 3a), but that they were also resistant to CmOOH-induced lipid peroxidation (a classic inducer of lipid peroxidation [25]), when compared to cells pre-adapted to 3.0% O_2_ (Figure 3b). To further explore the mechanism of the O_2_-dependent pre-adaptation of cells by which cells became resistant to subsequent killing by an inducer of lipid peroxidation, the effect of MSA addition (after O_2_ pre-adaptation but before CmOOH exposure) was assessed. MSA is an inhibitor of GPx, a key enzyme in the defence against lipid peroxidation [27]. When pre-treated with a range of MSA concentrations, cells which were pre-exposed to an environment of 18.6% O_2_ for 96 h were not sensitised to a subsequent treatment with 12.5 µM CmOOH. In contrast, MSA-pre-treated cells which had been pre-adapted to 3.0% O_2_ for 96 h were partially sensitised to a subsequent treatment with 12.5 µM CmOOH (Figure 3c), although the extent of the permissive effect of MSA toward sensitisation was limited. This suggests that the pre-adaptation of cells to a standard cell culture [O_2_] conferred resistance to subsequent oxidative stress-induced cell death and lipid peroxidation when compared to cells pre-adapted to 3.0% O_2_ for 96 h.

We next tested the antioxidant enzyme activities in A431 cells after pre-adaptation to 18.6% or 3.0% O_2_. Cells pre-adapted to 18.6% O_2_ for 96 h showed heightened enzymatic activities of CAT (Figure 4a) and GR (Figure 4c) compared to the activities measured in cells pre-adapted to 3.0% O_2_ for 96 h. There were no significant differences in the enzymatic activities of SOD or GPx in cells grown in the two [O_2_] conditions (Figure 4b,d). To determine whether the heightened CAT activity observed in cells pre-adapted to 18.6% O_2_ was accompanied by a concurrent increase in CAT protein expression, Western blot analyses were performed. We observed a 5-fold increase in the CAT protein expression (Figure 5a) in cells pre-adapted to 18.6% O_2_ compared to 3.0% O_2_. This was larger than the associated 1.3-fold increase in CAT enzyme activity. As CAT catalyses the degradation of H_2_O_2_, we had originally hypothesised that the mean concentration of H_2_O_2_ would be decreased in cells pre-adapted to 18.6% O_2_ due to the observed higher CAT levels. On the contrary, however, we found that cells pre-adapted to 18.6% O_2_ had a higher mean concentration of H_2_O_2_ than cells pre-adapted to 3.0% O_2_, as assessed by the Amplex Red assay (Figure 5b). These observations together suggest that a proportion of the CAT protein was inactive in A431 cells that had been pre-adapted to 18.6% O_2_.

CAT can, under some conditions, be permanently inactivated by persistent exposure to H_2_O_2_ [40,41,42,43]. However, the apparent inactivation of CAT in A431 cells pre-adapted to 18.6% O_2_ may involve multiple other factors in addition to H_2_O_2_-mediated inactivation. For example, CAT is also inactivated by superoxide (O_2_^●−^) and other reactive oxygen species [44,45,46]. In cells exhibiting a high steady-state concentration of ROS such as O_2_^●−^, the specific activity of CAT may be regulated by several mechanisms [47,48,49,50,51]. Nevertheless, we suggest that the elevated activities of CAT and GR are components of a modified cellular phenotype caused by the pre-adaptation of A431 cells to 18.6% O_2_. This altered phenotype demonstrates an increased resistance to H_2_O_2_-induced cell death compared to cells pre-adapted to 3.0% O_2_.

In the present study, glyceraldehyde 3-phosphate dehydrogenase (GAPDH) was used as a housekeeping protein in CAT Western blotting (Figure 5a). GAPDH contains a hypoxia response element in the *GAPDH* gene promoter [52]. However, in the present study, A431 cells were grown under physioxic conditions (3.0% O_2_); an [O_2_] which was insufficiently low to cause HIF-1α activation (Figure 2c). Thus, there was no statistically significant difference in the levels of GAPDH (as obtained from densitometric analysis) when comparing A431 cells pre-adapted to 18.6% O_2_ versus 3.0% O_2_.

To explore a potential molecular mechanism for the observed increases in antioxidant enzyme activities and protein levels, we next measured the levels of nuclear Nrf-2 in cells pre-adapted to 18.6% or 3.0% O_2_ for 96 h. Increased basal nuclear levels of Nrf-2 protein were observed in A431 cells pre-adapted to 18.6% O_2_ (which contained higher antioxidant enzyme activities) compared to cells pre-adapted to physioxia for 96 h (Figure 6). This is in agreement with a previous report from our laboratory [15], where A431 cells which had been pre-adapted to 18.6% O_2_ for 48 h exhibited higher expression levels of the mRNA that encodes Nrf-2 protein, and higher expression levels of the mRNA that encodes proteins whose transcriptional regulation is controlled by Nrf-2, compared to A431 cells pre-adapted to 2.0% O_2_ for 48 h. Whilst other researchers [17] have shown that the activation of Nrf-2 by electrophilic compounds is altered by pre-adaptation to 18.6% O_2_, we have made the novel observation that the growth of A431 cells in 18.6% O_2_ alone is sufficient to increase the basal protein levels of Nrf-2 without the addition of exogenous electrophilic activators of Nrf-2. In this context, cell culture under 18.6% O_2_ can be viewed as an inducer of Nrf-2 activation, at least in A431 cells. The knockdown of the *NFE2L2* gene (encoding Nrf-2 protein) sensitizes cells to H_2_O_2_-induced cell death [53]. As such, the observed increase in the levels of Nrf-2 in A431 cells pre-adapted to 18.6% O_2_ may explain, in part, why these cells exhibit resistance to H_2_O_2_-induced cell death. However, the levels of Nrf-2 were only measured after a 96 h pre-adaptation to 3.0% or 18.6% O_2_. Whilst it appears that there is a relationship between increased Nrf-2 levels and the observed resistance to H_2_O_2_-induced cell death in cells pre-adapted to 18.6% O_2_, a definitive conclusion regarding Nrf-2’s direct involvement in this resistance is confined to the 96 h pre-adaptation time point. Additionally, as the Nrf-2 protein levels were only measured at the 96 h pre-adaptation time point, the time taken for Nrf-2 to lose activity upon the exposure of A431 cells to 3.0% O_2_ is yet unknown.

Why are the basal protein levels of Nrf-2 increased in A431 cells pre-adapted to 18.6% O_2_ for 96 h? Keap-1, a Cullin-3 E3 ubiquitin ligase, normally targets Nrf-2 for proteasomal degradation [54]. However, Keap-1 may act as a sensor for H_2_O_2_ through specific cysteine residues [54]. A persistent (though modest) increase in the steady-state concentration of H_2_O_2_ in A431 cells that have pre-adapted to 18.6% O_2_, compared to cells in an environment of 3.0% O_2_ (Figure 5b), may activate Nrf-2 through these H_2_O_2_-sensitive Cys residues in Keap-1. We propose that the redox homeostasis of A431 cells is fundamentally altered by long-term cell culture in 18.6% O_2_, compared to the same cells grown long-term in physioxia, due to it increasing the activity and levels of Nrf-2-controlled antioxidant defence systems. The increased level of nuclear Nrf-2 protein was associated with an increase in the levels and enzymatic activities of CAT and GR, these being encoded by antioxidant genes whose transcription is regulated by Nrf-2 [55]. Yet, among the enzymes whose corresponding genes are transcriptionally regulated by Nrf-2, not all showed heightened activity in A431 cells pre-adapted to 18.6% O_2_. Thus, the activity of SOD and GPx were unaffected by pre-adaptation to 18.6% O_2_ compared to 3.0% O_2_.

Why then do some Nrf-2-target enzymes exhibit increased activity after pre-adaptation to 18.6% O_2_, but not others? Previous research [17] has noted that the pre-adaptation of human umbilical vein endothelial cells to 18.6% O_2_ increased their protein expression of certain Nrf-2-target genes such as NQO-1, but not haem-oxygenase 1. One protein which may be involved in the selective transcription of Nrf-2 gene targets is Bach-1, which inhibits the transcription of about 200 genes with an Nrf-2-binding motif [55,56,57,58,59]. The inhibition of Nrf-2 by Bach-1 could explain why the antioxidant enzymes SOD and GPx, partially regulated by Nrf-2, do not demonstrate increased activity in A431 cells pre-adapted to 18.6% O_2_. As Nrf-2 regulates the antioxidant stress response [55,56,57,58,59], the altered activity of these proteins in cells pre-adapted to 18.6% O_2_ may impact on subsequent attempts to induce oxidative stress in vitro. H_2_O_2_ is a classical inducer of oxidative stress [60] and is used to induce cell death in vitro [3,4,5,6,7,9,11,12,61,62,63,64,65,66], and to test the efficacy of antioxidant compounds. However, high concentrations of H_2_O_2_ (>100 µM) are often required to elicit oxidative damage in cells grown using in vitro cell culture systems [66]. These concentrations of H_2_O_2_ are higher than the [H_2_O_2_] range in vivo [67,68]. The use of high concentrations of H_2_O_2_ to study cellular effects such as cell death has been described as ‘The Thermonuclear Attack Model’ by Forman et al. [69]. The biological significance of results obtained using such high H_2_O_2_ concentrations should be considered with great care. The explanation for why these high H_2_O_2_ concentrations are often used by researchers to induce cell death in vitro is multifaceted. The present study suggests that the effectiveness of H_2_O_2_ as an inducer of cell death in squamous cell carcinoma cells may be, in part, dependent on the [O_2_] that mammalian cells are pre-adapted to prior to their subsequent treatment with H_2_O_2_ (Figure 1c). Our H_2_O_2_ concentration–response tests showed an increase in the LC_50_ of H_2_O_2_ from 0.6 mM to 1.1 mM when comparing cells pre-adapted to 3.0% O_2_ for 96 h versus cells pre-adapted to 18.6% for 96 h, respectively. However, a high concentration of H_2_O_2_ (>500 µM) was still required to kill even the cells pre-adapted to physioxia for 96 h. In essence, to detect oxidative damage upon the addition of H_2_O_2_ to mammalian cells grown in vitro at 18.6% O_2_, a high enough concentration of H_2_O_2_ must be added to overcome cellular antioxidant defences. However, whilst this present work implicates an increased Nrf-2 activity in A431 cells pre-adapted to 18.6% O_2_ as a mechanism to explain their cellular resistance to H_2_O_2_-induced death, this was not demonstrated experimentally using molecular approaches (e.g., *NFE2L2* gene knockdown via siRNA). Therefore, further research is needed to confirm the role of increased antioxidant defences in the resistance to H_2_O_2_-induced cell death of A431 cells pre-adapted to 18.6% O_2_.

Whilst the [O_2_] used in standard cell culture may explain, in part, why cells grown in vitro resist subsequent H_2_O_2_-induced oxidative damage, it is likely not the only reason. Other factors clearly impact the resistance of cells to H_2_O_2_-induced cell killing. Fe^2+^ in the cell culture medium, and in FBS, may protect against cell damage caused by the addition of exogenous H_2_O_2_ by catalysing the extracellular production of the hydroxyl radical (^●^OH) within the cell culture medium, leaving less H_2_O_2_ available to diffuse into the cells [70,71,72,73,74,75,76,77,78,79]. The issue of the pro-oxidant state of commonly used cell culture growth media (e.g., DMEM) has been highlighted by B. Halliwell [74]. Other issues which may alter the H_2_O_2_ induction of oxidative stress include monolayer versus organoid cell cultures [80], co-culture versus monoculture [81], the glucose concentration in cell culture mediums [82], cell line genetic drift [83], and [O_2_] gradients [84,85]. In addition to the many previously mentioned contributing factors which may affect H_2_O_2_ reactivity in standard cell culture investigations, the present data support the observations [15,16,17,18,19,20,21] that redox-active compounds—when tested on mammalian cells pre-adapted to 18.6% O_2_—produce quantitatively different effects compared to cells pre-adapted to physioxia.

This present investigation utilised a single transformed cell type (A431 cells). Therefore, it is not possible to generalise as to whether the resistance of A431 cells to H_2_O_2_-induced cell death, once the cells have been pre-adapted to 18.6% O_2_, extends to other cell types of a phenotype close to A431 cells (e.g., epidermal keratinocytes). Because we observed H_2_O_2_-induced lipid peroxidation in A431 cells (a key marker of ferroptosis [86]), we cannot rule out the possibility that the cells underwent ferroptotic cell death (as opposed to apoptosis) in our study. H_2_O_2_ has been shown to induce ferroptosis in rat glioma cells [29] and primary cardiomyocytes [87]. In the literature, it has been widely held that H_2_O_2_-induced cell death is due to apoptosis [3,4,5,6,7,9,11,61,62,63,64,65]. However, the relatively recent discovery of the ferroptotic pathway of cell death [88] necessitates a re-evaluation of the earlier literature. Additionally, H_2_O_2_ reacts with copper-containing proteins (e.g., caeruloplasmin), resulting in the release of copper ions which catalyse ^●^OH formation through a Fenton-type reaction [89,90]. As such, cuproptosis—a recently discovered copper-dependent form of cell death [91]—could be involved in the cell death observed in the present study. Both these possibilities need further investigation. However, whether the observed cell death was by apoptosis, ferroptosis, or cuproptosis, this does not alter the basic observation reported here that A431 cells pre-adapted to 18.6% O_2_ are resistant to H_2_O_2_-induced cell death.

## 5. Conclusions

A431 cells which had been pre-adapted to 18.6% O_2_ for 96 h were resistant to the cell death induced by their subsequent exposure to added exogenous H_2_O_2_ compared to cells pre-adapted to physioxia (3.0% O_2_). Cells which have been pre-adapted to 18.6% O_2_ for 96 h may, in part, be resistant to H_2_O_2_-induced cell death because of their increased protein expression and activities of certain antioxidant enzymes (GR and CAT). Such changes may be associated with the observed heightened activation of Nrf-2. We propose that the resistance to H_2_O_2_-induced cell killing exhibited by A431 cells pre-adapted to 18.6% O_2_ (when compared to cells pre-adapted to 3.0% O_2_) is artefactual, and is caused by culturing the cells long-term in a non-physiological [O_2_] prior to H_2_O_2_ exposure.

## Data Availability

The data presented in this study are available in the article and Supplementary Materials.

## Supplementary Materials

The following supporting information can be downloaded at https://www.mdpi.com/article/10.3390/antiox13030269/s1, Figure S1: schematic representation of the design of the ‘Klip-lock’ container and a graph showing the time course of the decrease in oxygen concentration during gassing of the Klip-lock container.
