# Supplementary material for: Cellular Pre-Adaptation to the High O2 Concentration Used in Standard Cell Culture Confers Resistance to Subsequent H2O2-Induced Cell Death
Source: Antioxidants (Basel). 2024 Feb 22;13(3):269. doi: 10.3390/antiox13030269 (PMC10967602; doi:10.3390/antiox13030269)
Supplement: Supplementary file 1 [file antioxidants-13-00269-s001.zip › antioxidants-2810730-supplementary.pdf]

Supplemental material

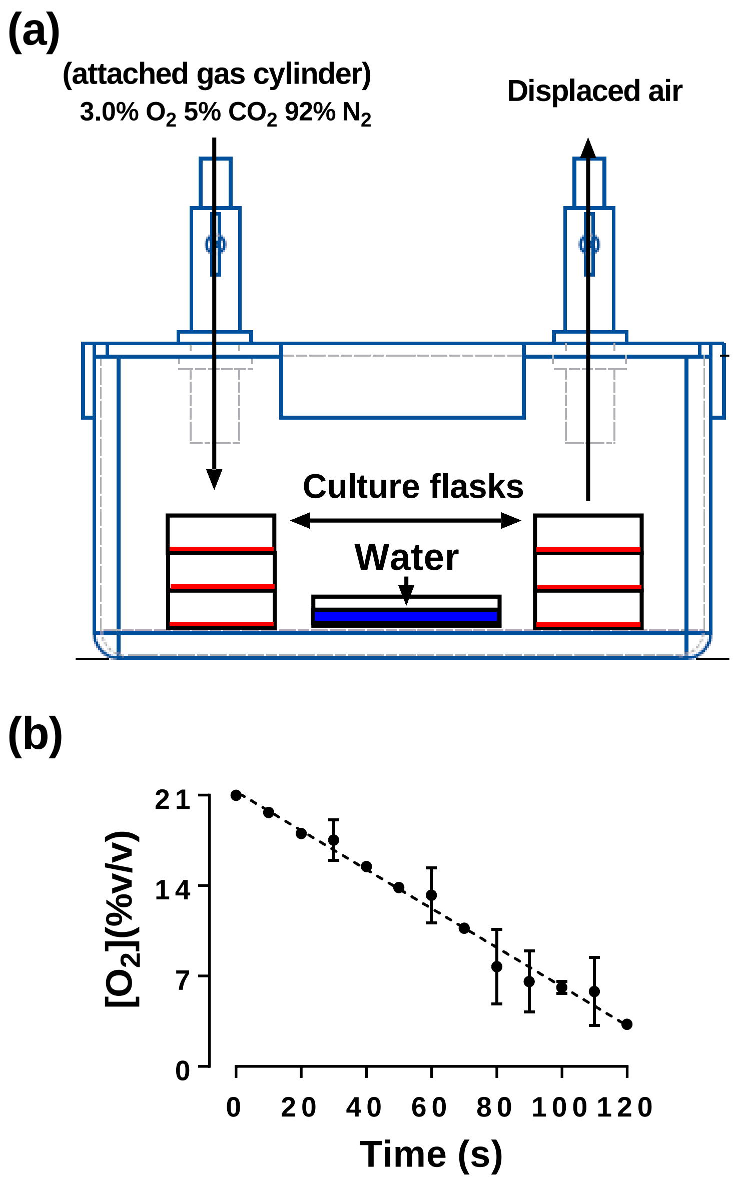

Supplemental Figure S1: Schematic representation of the design of the 'Klip-lock' container and a graph showing the time-course of the decrease in oxygen concentration during gassing of the Klip-lock container. A 'Klip-lock' box containing an O_2 gas detector was purged of atmospheric gas by attaching a pre-mixed gas cylinder (3.0% O_2, 5.0% CO_2, 92.0% N_2; DDRC Healthcare) to the inlet valve. The inlet and outlet valves were then opened, and the gas flow was turned on. Over time, gas was displaced through the outlet valve until the [O_2] reached 3.0% v/v. Once the measured O_2 gas environment had stabilised (~2 min), both valves were closed. To generate a humidified environment, a petri 60 mm Petri dish was filled halfway with autoclaved water. The container was then transferred into a physioxia hood set at 37 °C. **Panel (a)**, 'Klip-lock' box used for the growth of cells in 3.0% O_2. **Panel (b)**, the decrease in [O_2] in 'Klip-lock' containers during the gassing procedure using pre-mixed gas. Data in panel (b) are presented as the mean ± SD, n = 3. Where error bars are not visible, this is because the error bar is smaller than the size of the data point. The dashed line represents

the best fit line generated by linear regression ($R^2 = 0.94$). The image in panel (a) was kindly produced using CREO parametric software (Parametric Technology Corporation, Boston, USA) by L. Jordan (affiliated with Atlantic Technological University, Republic of Ireland).
